# Probing Structural Perturbation of Biomolecules by Extracting Cryo-EM Data Heterogeneity

**DOI:** 10.3390/biom12050628

**Published:** 2022-04-24

**Authors:** Kira DeVore, Po-Lin Chiu

**Affiliations:** School of Molecular Sciences, Biodesign Center for Applied Structural Discovery, Arizona State University, Tempe, AZ 85287, USA; kdevore1@asu.edu

**Keywords:** single-particle cryo-EM, heterogeneity, molecular dynamics, deep learning, molecular dynamics flexible fitting, image classification

## Abstract

Single-particle cryogenic electron microscopy (cryo-EM) has become an indispensable tool to probe high-resolution structural detail of biomolecules. It enables direct visualization of the biomolecules and opens a possibility for averaging molecular images to reconstruct a three-dimensional Coulomb potential density map. Newly developed algorithms for data analysis allow for the extraction of structural heterogeneity from a massive and low signal-to-noise-ratio (SNR) cryo-EM dataset, expanding our understanding of multiple conformational states, or further implications in dynamics, of the target biomolecule. This review provides an overview that briefly describes the workflow of single-particle cryo-EM, including imaging and data processing, and new methods developed for analyzing the data heterogeneity to understand the structural variability of biomolecules.

## 1. Introduction

The revolution in cryogenic electron microscopy (cryo-EM) has dramatically advanced knowledge in the field of structural biology [1,2]. Technical advances in hardware improvement, sample preparation, and algorithms for data analysis have pushed the limit of cryo-EM imaging in biological applications. Thanks to the fast image acquisition of the direct electron detector (DED) camera, the electron images can be recorded in a time series as a molecular movie [3,4,5]. Massive image processing tasks can be efficiently completed by increasing computing power, making the image reconstruction workflow more efficient. For single-particle cryo-EM, it has now become a routine to collect thousands of electron movies per day on a transmission electron microscope (TEM) and obtain an initial three-dimensional (3D) model from a computer workstation within a few hours or days. The generated high-resolution structural information of biomolecules dramatically expands our understanding of the molecular mechanisms that benefit the fields of biology and medicine.

In a typical single-particle cryo-EM workflow, the protein molecules are rapidly frozen and present multiple states and orientations in thin vitreous ice. The electron scattering by the proteins forms an image by the electromagnetic lenses and is recorded on a detector. Individual molecular images are then used to reconstruct the 3D Coulomb potential density of the target molecule [6]. The reconstruction requires accurate Euler angle assignments on individual two-dimensional (2D) particle images and an appropriate interpolation scheme for 3D back projection [6,7]. High-resolution details of a protein structure, such as residue side chains or ligands, could emerge by averaging a large number of aligned images of the same entity, because the averaging process flattens the noise and increases the signal-to-noise ratio (SNR). However, in some cases, the same averaging procedure does not generate any high-resolution structural details (possibly due to small movements of side chains) or will resolve the protein density at a very low contour level (possibly due to a disordered structure). It could be that the structural variations recorded in the 2D projections smear the densities in the moving trajectory or flatten out the featureless densities. Thus, a 3D reconstruction can be seen as a back-projected average of 2D particle images with their structural variabilities flattened.

One of the challenging problems in analyzing cryo-EM data is that the image has a low SNR. An efficient and effective algorithm will be needed to extract the data heterogeneity related to the molecule’s structural variability. Because biological molecules are sensitive to electron radiation damage, one has to use a lower electron dose to image the specimens to preserve the structural integrity [8,9,10,11]. The signal content of the object is then compromised by the low imaging dose, which limits the signal level that can be extracted from the noise. One can increase the particle data size to increase the SNR by averaging. Recently, new strategies of cryo-EM data collection have enabled generating a large particle dataset within a reasonable timeline [12]. Newly developed algorithms, including machine learning-based approaches, open the possibility of analyzing the molecular motions that are captured in cryo-EM images, such as ribosomes [13,14,15], spliceosomes [13,16,17,18], and G-protein coupled receptors (GPCRs) [19,20,21,22].

This review will first overview the typical processing workflow of single-particle cryo-EM. Next, we will discuss typical sources of heterogeneity within a dataset and list the algorithms used to analyze the image variance by reviewing the principles of conventional multivariate statistical analysis (MSA), optimizations using maximum likelihood (ML), and Bayesian parameter estimation. We will then introduce machine learning approaches to derive a dynamical model from the cryo-EM data. The last part will list some current methods used to analyze the cryo-EM heterogeneity or prepare the cryo-EM sample at different time points, so-called time-resolved cryo-EM. The extracted information from cryo-EM data heterogeneity can provide a knowledge framework about biomolecular dynamics, which may implicate its function and improve the understanding of its working mechanism.

## 2. Typical Workflow of Single-Particle Cryo-EM

**Figure 1:**
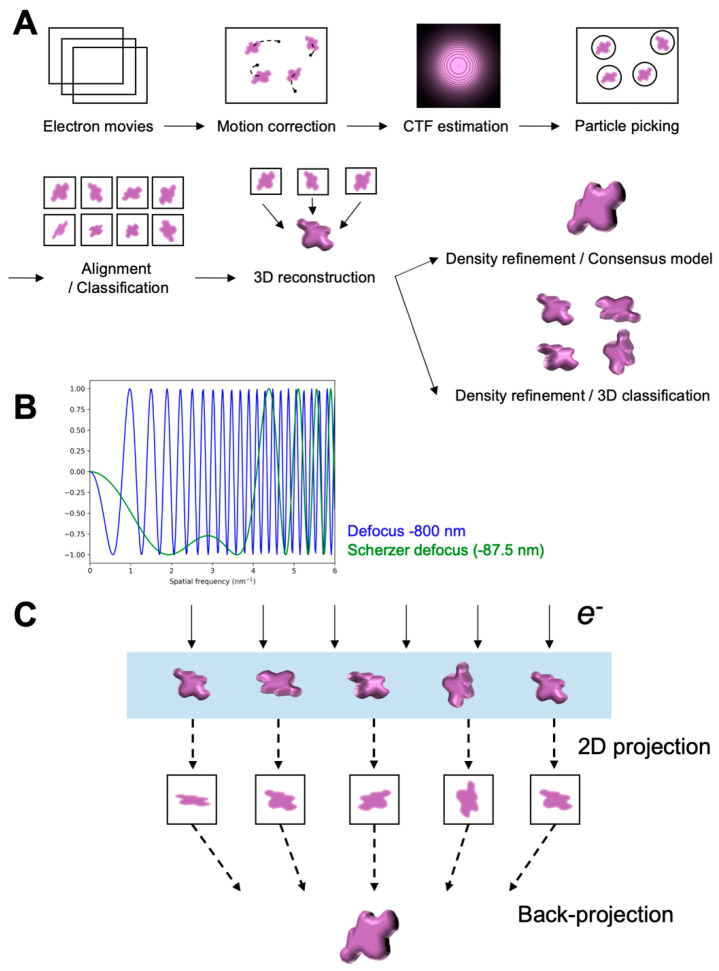
Schematics of the single-particle cryo-EM processing workflow. (**A**) General processing workflow for single-particle reconstruction workflow. (**B**) One-dimensional plot of the contrast transfer function (CTF) versus spatial frequency. Curves are calculated using an accelerated voltage of 300 keV and a spherical aberration coefficient (*Cs*) of 2.7 mm. Blue and green curves are for the defocus at −800 and −87.5 nm (Scherzer defocus), respectively. (**C**) Schematics of 2D projections from an imaged object and a 3D back-projection from the 2D projections. Light blue represents thin ice that embeds the particles in different orientations.

A typical single-particle cryo-EM workflow begins with a biochemically homogeneous protein sample (Figure 1A). To preserve the sample in a hydrated state in the EM column, one can embed the sample in an anti-freezing agent, such as trehalose [23], or vitrify the sample by fast freezing into a cryogen, such as liquid ethane or liquid propane, so-called plunge freezing. Vitrification results in amorphous ice formation below the glass transition temperature and prevents crystalline ice from damaging the proteins [23]. Additionally, because the vitrified sample is in a hydrated state, the preserved structure is close to its native condition. The idea of plunge freezing was initially developed by the 2017 Noble Prize laureate Jacques Dobuchet in the 1980s. It was shown that pure water could be vitrified and used for EM imaging on radiation-sensitive materials [24]. The cryogenic biological sample of adeno-associated viruses was first demonstrated structurally intact and feasible for cryo-EM imaging [25].

A biological specimen is composed of light elements, which can be seen as a weak-phase object that only modulates the phase of the incoming wave [26,27]. Because the intensity variations of a weak-phase object are minimal, the image contrast is minimal in focus. Because one can change the focal plane by adjusting the objective lens strength, this allows for the modulation of the contrast transfer function (CTF). The typical use for imaging a biological specimen is to introduce a defocus to enhance the contrast in the lower spatial frequency region in a non-linear manner [28,29]. However, defocusing causes a high contrast transfer oscillation in the high-frequency region and reduces the spatial coherence, compromising the quality of the phases in the high-frequency region for the subsequent image reconstruction (Figure 1B) [28,29].

Thanks to the fast frame acquisition and high detective quantum efficiency (DQE) of the DED camera, it opens wide possibilities for preserving the structural information of the imaging targets in both spatial and temporal domains [30,31]. Compared to conventional detectors, such as photographic films or charge-coupled device (CCD) cameras, the DED camera directly detects the electrons and allows for a counting mechanism, which has a high-frequency response in the high-resolution region [32]. Additionally, upon electron beam exposure, the specimen moves due to the release of its mechanical strain, resulting in so-called beam-induced movements, which deteriorate the image quality [33,34,35,36,37]. The DED camera is capable of fast image acquisition to enable the recording of a movie, which can be used to register the translational movements between each frame and obtain an average that improves the SNR of the object [32,38,39]. Furthermore, more improvements in the image quality due to the enhanced capabilities of the DED camera have been pushing the limits of cryo-EM, such as super-resolution [31] and dose weighting [38].

Molecular projections are selected from the electron images and are used to calculate a 3D Coulomb potential reconstruction of the target molecule [6]. Before reconstructing a 3D density volume, the 2D particle projections are aligned to a common origin. In addition to the conventional cross-correlation approach, the maximum-likelihood method is generally used to find the parameters, such as poses, that maximize the maximum likelihood estimates (MLE) [40,41,42]. In this framework, the expectation-maximization (EM) algorithm postulates the Gaussian probability models and calculates the MLE iteratively [42,43]. One of the disadvantages of the EM algorithm is the occurrence of the convergence to the nearby maximum, rather than the global maximum. Approximate Euler angles are initially assigned to individual 2D particle projections, and along the assigned projection direction, the 2D image intensities are used to integrate and back-project into a 3D volume (Figure 1C) [44]. The back-projection algorithm is based on the central projection theorem, which states that the Fourier transform of a 2D object projection is one slice of the 3D Fourier transform of the object when the Ewald curvature is flat [45]. The assigned Euler angles can be iteratively updated and improved by comparing the newly generated reference density. However, the Euler angle assignment for each particle projection could be challenging given that the SNR of cryo-EM data is extremely low. Therefore, using an unvalidated reference model, or attempting to select particles from a low SNR dataset based on this model, could lead to a biased map that largely resembles the model [46]. Thus, the image processing and the reconstructed model will need rigorous validation to ensure minimal reference bias. The resolution of the final 3D density map is cross-validated using the criteria of the golden Fourier-shell correlation (FSC) [47].

False positives are always generated when identifying particles in an image [48]. To minimize the false positives, the typical approach is to iteratively perform 2D image classification and select the classes with discernible molecular features [48]. However, due to the low SNR cryo-EM image and the error rate of the classification procedure, many particle images could be imprecisely aligned or wrongly assigned, leading to the removal of presentable particle projections. This could down-weight the particle projections in the intermediate states and flatten the final class averages. Therefore, the conventional curation approach may need to be reexamined to prevent the removal of true particle images and preserve the information for extracting the structural variability of the target molecule.

## 3. Sources of the Cryo-EM Image Data Heterogeneity

Biomolecules perform their function by changing their structures. The structural flexibility of a protein varies across different forms and mostly depends on its physical nature. However, molecules are always mobile because of thermal fluctuations [49]. When triggered by an environmental stimulus or a ligand, a biomolecule can conduct a structural change, globally or locally, and transition to a different energy state to perform its function [50]. Therefore, the proteins in vitreous ice may present different conformational states in an aqueous solution, which can be captured by cryo-EM imaging. The molecular simulation study showed that the fast-cooling process prevents the molecules from crossing the energy barriers, leading to the entrapment of molecules in a conformational ensemble [51]. A consensus 3D density map, a map back-projected using all of the particle images, could be used to estimate the signal certainties or voxel variances of the structural flexibility of the target molecule [52,53,54]. Local resolution estimation is the approach used to identify the global or local mobility of a protein. However, since the consensus map only presents a static view of the molecule, the estimated map does not contain the magnitude or the direction of motion of the protein local domains.

**Figure 2:**
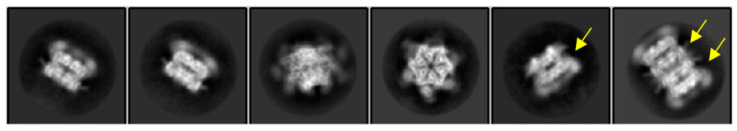
Two-dimensional (2D) class averages of cryo-EM particle images show the compositional heterogeneity. p97 R155H mutant complex [55]. Box side length is 374 Å. Yellow arrows indicate one single protein complex.

When mixing protein complex components in vitro for cryo-EM study, the molecules may form homo- or hetero-oligomers. Depending on the stability of the interactions between proteins, the ensemble may contain different molecular species, leading to compositional heterogeneity in the particle population. Conventional 2D image classification could be used to sort out this compositional heterogeneity, for example, the p97 mutant complex in dodecameric and hexameric forms [55] (Figure 2). In some cases, if the angular distributions of individual molecular species are even for reconstructing their 3D volumes, the particle proportions could help understand the complex formation for the binding reaction.

**Figure 3:**
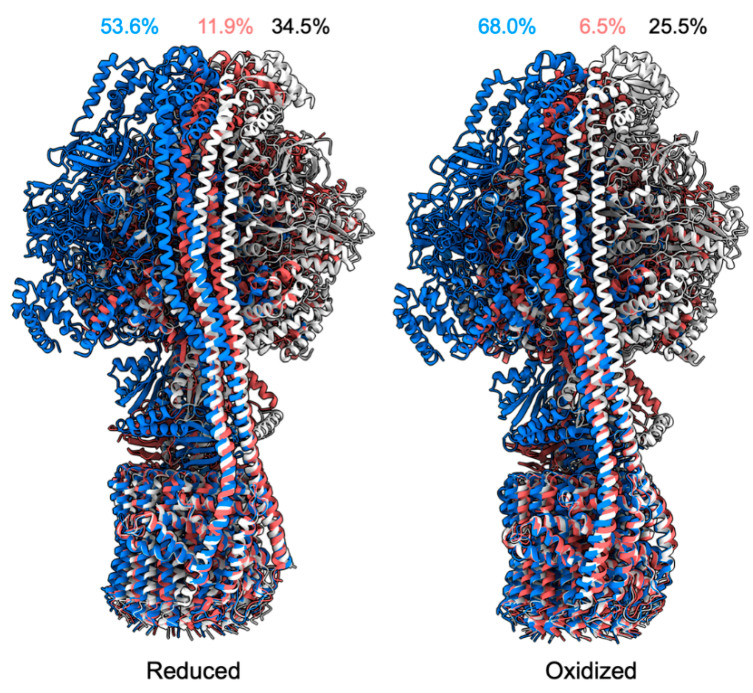
Redox states of chloroplast ATP synthase [57]. Blue, white, and salmon are different rotary states of the ATP synthase. Percentages labeled are the particle proportions within those in individual redox states.

According to the dynamic nature of biomolecules, conformational transitions can be discrete, continuous, or disordered for their function. For instance, ATP synthase is a molecular motor complex that generates or consumes the energy currency, ATP, for sustaining a cell’s life, which has been extensively studied using single-particle cryo-EM [56]. The cryo-EM reconstruction of ATP synthase typically generates three distinct states, which are in equilibrium in an aqueous solution [56]. Three different structures present the central stator rotating in various stages, with different proportions of particles in the population. Moreover, chloroplast ATP synthase activity can be modulated by the redox state of the central stator [57,58]. Interestingly, the three distinct rotating states could be resolved in their equilibrium states, but the proportions of the three states were different, implying the structural change in the complex changes the energy profiles of the three states [57] (Figure 3).

Some biomolecules move continuously and do not exhibit distinct structures in the solution, which is not trivial to be categorized using conventional image classification to identify individual conformations on low-SNR cryo-EM images. In the cases of proteins that are disordered and do not present a structural feature, it is usually challenging to reconstruct high-resolution densities in these flexible regions by the conventional averaging approach.

## 4. Conventional Approaches to Study Cryo-EM Data Heterogeneity

One of the goals of single-particle reconstruction is to seek an optimal solution for Euler angular assignment to the 2D particle projection for a high-resolution 3D back-projection [7]. To obtain the dynamic information of a target molecule, the imaging will require capturing sufficient 2D projection data to present the structures in different states or different time points. The subsequent image classification scheme will be carried out to detect the structural differences and group the images into different classes in either a supervised or unsupervised manner. The variation between the 3D reconstructions from different groups could provide a dynamic view of the target protein.

### 4.1. Conventional Multivariate Statistical Analysis (MSA)

Multivariate statistical analysis (MSA) was first introduced to identify different particle views and average the class members to improve the SNR of the images of the negatively stained samples [59]. The fundamental concept is to find the orthogonal axes that can best categorize the image data into several groups and register individual particle images in a hyperspace [59,60]. This will require reducing the image dimensionality into several components, so-called dimension reduction [61]. Principal component analysis (PCA) is commonly used to determine the principal axes and eigenvectors. Correspondence analysis (CA) has also been used to create orthogonal axes and define the data in a relative manner, rather than the absolute Euclidean distance as in PCA [62]. Note that the efficiency of MSA is subjected to the SNR of the data and is highly dependent on the image alignment accuracy, which could be generally improved using an iterative method [59,63]. More EM image analyses using MSA can be found in the following works [59,64,65,66].

*k-*means clustering is the most used approach to categorize particle image data, which finds the centroids by starting random seeds and updating them iteratively until the optimal centroids are found [67]. An iterative algorithm could improve the clustering, but it could sometimes converge to a local minimum, complicating the reproducibility [48]. The number of *k* seeds is critical to the success of the classification, which is usually determined empirically. Additionally, *k-*means clustering is subjected to the SNR of the data. In the case of low-SNR data, the clustering algorithm usually produces clusters of similar sizes and does not calculate the centroids accurately, biased by the noise level. Moreover, suppose the number of seeds, *k*, is larger than the number of the representative classes in the data; in that case, some classes will collapse without any members assigned or the particles will be wrongly assigned into dominated classes, lowering the efficiency and accuracy of the clustering.

To mitigate the disadvantage of *k-*means clustering and prevent the collapse of the classes, some approaches have been developed to improve *k*-means clustering [68,69,70,71,72]. One of them is the iterative stable alignment and clustering (ISAC), which was proposed to utilize the Equal-Size group *k*-means (EQK-means) method that equalizes the size of individual classes to prevent the class from collapsing. The classes are then tested with reproducibility to stabilize the classification [68]. The ISAC method is usually applied to 2D image analysis and has the advantage of sorting out the classes that are rarely found in the particle population. Because the algorithm rigorously tests the reproducibility of the particle assignment to the classes, it may take a longer time to complete the classification when the data size is large.

The classification of the 3D volumes could be performed in a supervised manner using projection matching or MSA if the structures of multiple states are known [73,74]. The algorithms iteratively update the assigned Euler angles and classes of the 2D particle images by comparing the projections of all input 3D volumes. Because prior knowledge of structures is required, the results are strongly dependent on the model and may be subjected to reference bias if the SNR of the data is low or proper cross-validation is not implemented [75].

### 4.2. Regularized Likelihood Approach

Maximum likelihood (ML) estimation has been long used in crystallographic refinement and single-particle EM in the field of structural biology [41,76]. In single-particle EM, Provencher and Vogel first used the ML algorithm in reconstructing the 3D volumes of biomolecules [77]. Sigworth implemented the ML algorithm in 2D image analysis and proposed the ML optimization for use in single-particle reconstruction [41]. Along with the same concept, Scheres and his coworkers developed a new ML3D (3D maximum likelihood) algorithm to classify single-particle image data for 3D reconstructions [78].

The ML3D algorithm is an expectation-maximization (E-M) algorithm, which seeks structural models that maximize the likelihood of the data by the marginalization over particle orientations and class assignments in an iterative manner [42,79]. Because the approach could converge in a local minimum, it is suggested to begin with a consensus model that is representative of the data when using ML3D [42]. Additionally, the ML3D algorithm uses a multi-reference refinement scheme, which may introduce some extents of reference bias. This will require a validation step by refining independent groups of the data. The current ML approach uses an iterative algorithm to obtain the solution, but this does not ensure convergence and may have a high unnecessary computation cost. On the other hand, the representable number of clusters in the data will need to be determined empirically. It may take a very long time computationally to optimize the class number, *k*. When *k* is overdetermined, the generated classes will not be representable for the data, whereas when *k* is underdetermined, the heterogeneous features may be diminished in the class averages. Additionally, because the class number *k* is assigned as an integer, presumably describing discrete states in the particle ensemble, it would be trivial to resolve the structural differences within the data or map the trajectory by connecting the resolved 3D class averages if continuous molecular movement is present.

In some cases, the flexible part is only a local portion of the molecule. One can use a smaller mask to limit the density refinement only carried out in the region of interest, improving the accuracy of the alignment and classification [80]. Furthermore, this local refinement could be improved by subtracting the densities that are not of interest, so-called signal subtraction [81]. However, the success of this local refinement procedure is subjected to the size of the selected or masked region, the angular distribution of the particle projections, and the SNR of the data. If the size of the selected region is too small, the accuracy of the image alignment may be compromised. When the target molecule has a concerted movement involving more than one region or domain, one can use a similar strategy to apply to multiple sites of the density and analyze their movements, so-called multi-body refinement [13].

Cossio and Hummer detailed the current methods that adapt the likelihood approach for analyzing cryo-EM data heterogeneity [82].

## 5. Continuous Structural Heterogeneity Derived from Cryo-EM Data

### 5.1. Covariance Matrix Estimation

The conventional way to analyze the data heterogeneity is to determine the top principal components or eigenvectors, which are mostly associated with molecular variabilities. However, it becomes challenging when resolving heterogeneity in 3D, but the data are provided in 2D. A recent study proposed a new covariance matrix estimator to analyze the relationships between unknown variables in 2D data and use this estimator to resolve the 3D heterogeneity [83]. The advantage of this approach is that no knowledge about prior distribution or data ranks is required. However, the SNR is still the limiting factor to its effectiveness. More parameters, such as the CTF, may be required to take into consideration for qualitative comparison with traditional PCA in high dimensions.

### 5.2. Hyper-Molecules

The concept of the “hyper-molecules” is to add high-dimensional variables and map the continuous structural heterogeneity with a Bayesian formulation and use the Markov chain Monte Carlo (MCMC) algorithms to sample the posterior distribution without the need for normalization [84,85]. The advantage of the MCMC approach is to reduce the computational cost and have the capability to expand in combination with other algorithms to improve the performance. The algorithm has been applied on a simulated dataset to demonstrate its feasibility.

### 5.3. 3DVA (3D Variability Analysis) Approach

A 2D cryo-EM particle projection only presents a partial observation of the 3D structure of the object, and the structural information contained in the image is altered by the imaging. For example, the quality of the image phases depends on the CTF modulation and is also subjected to the noise level. Therefore, when conventional PCA is used to analyze the data for molecular motions, the number of dimensions may need to be increased, which requires numerous amounts of parameterization and complicates the computation. To overcome this drawback, the 3DVA (3D variability analysis) algorithm uses E-M optimization for probabilistic PCA (PPCA) to seek linear subspace models for single-particle image data [86,87,88,89,90]. PPCA generalizes the conventional PCA, when the covariance of the noise is very small. The probabilistic formulation provided by PPCA could improve the efficiency of finding the ML estimates from the principal components. To use the 3DVA algorithm, a precise image alignment for the consensus model is required. It resolves the data heterogeneity better when analyzing the data in a limited resolution range, which could be related to a better SNR level in the low spatial-frequency region. If the size of the flexible moiety is small, 3DVA still has its limitation to extract the variability components to accurately model this moiety that partakes in dramatic distance changes [90].

### 5.4. CryoDRGN

CryoDRGN (Deep Reconstructing Generative Networks) was designed to probe continuous 3D structural heterogeneity using a generative deep-learning approach [16]. The algorithm encodes the 2D projection data into a latent space using a variational autoencoder (VAE) and decodes slices of a series of 3D reconstructions. A VAE uses a probabilistic formalism to describe the data in a latent space, which can decode the generative models with continuity, or in a smoother way. The early version of the program uses the poses that are optimized for a consensus reconstruction [91], and the latest version includes the optimization of the poses while encoding the data into a latent space [91]. The program provides graphic representations of the projection data via either PCA or UMAP (uniform manifold approximation and projection) [16]. CryoDRGN has been shown to resolve motions of large biomolecular complexes, such as the 80S ribosome and spliceosome complex [16].

Because CryoDRGN adapts a deep neural network framework, the success of resolving continuous motion will be inherently limited by the parameters embedded within its construction. The latent space may need to be optimized empirically [16]. Additionally, overfitting may occur when the data SNR is low or the number of parameters is underdetermined. CryoDRGN utilizes latent space as a form of data reduction, which could be useful for determining the underlying flexibility of molecules but may not implicate any physical properties of the molecules, such as the energy landscape. Additionally, given the diversity of origin regarding heterogeneity within single-particle cryo-EM datasets, this latent space approach may not be representative for all forms of heterogeneity.

## 6. Mapping Energy Landscape from Cryo-EM Data

The domains within the protein do not move randomly but are constrained by neighboring domains or surrounding environments, and these movements, or conformational changes, usually accompany energy transitions. The proportions of the conformational states follow the Boltzmann distribution, but it is not trivial to reconstruct the energy landscape with individual conformational states from 2D cryo-EM projections, which are mostly noisy with a preferred Euler angle distribution.

Instead of conventional image classification, ManifoldEM was designed to map an energy landscape from the raw image data [92,93,94]. The method uses particles assigned with the same Euler angles and projects them onto a hyperspace. The particle projections in similar conformational states will more densely populate within a particular manifold in the hyperspace. The advantage of this method is that no preliminary classification is required, and it uses raw image data for the computation. Hence, this method will need a large number of datasets. The program showed promising results for extracting conformational variabilities from single-particle cryo-EM datasets of the 80S ribosome and ryanodine receptor, supported by atomistic simulation results as the ground truth [93].

In a deep-learning framework, AlphaCryo4D uses an energy-based particle-voting algorithm to encode 3D feature densities onto a Boltzmann-weighted embedding landscape [95]. Through searching local energy minima along a minimum-energy path, the 3D densities at transition states could be extracted and reconstructed [95]. This has been used to elucidate the transition of ubiquitin binding on the human 26S proteasome complex at different proposed functional states [95]. However, the method may not accurately analyze a small dataset, which may undersample the particle projections for 3D reconstruction at local minima [95].

The cryo-BIFE (cryo-EM Bayesian inference of free-energy profiles) method extracts the free energy profile using a lower dimensionality with collective variables (CV) involving Bayesian inference [96]. The method investigates the parameters of the collective variable, allowing to uncover the pertinent conformational states of a molecule. The method has been applied to calculate the free energy profile of TMEM16F ion channels and successfully showed two states that are bound with and without calcium ions [96].

## 7. Hybrid Approaches with Molecular Dynamic Simulations

The image quality resulting from averaging depends on the flexible nature of the target molecule, and it is common that the resolution of the cryo-EM density is not sufficient for accurately modeling the atoms globally or locally. If the target molecule is highly flexible, it may not be trivial to determine a high-resolution structure using the typical single-particle cryo-EM approach since the density of the mobile region will be flattened due to averaging. However, the low-resolution cryo-EM density provides some positional uncertainties for the atoms, which could be useful in understanding the regional flexibility. Low local resolution can be an indicator of a highly flexible region. If the cryo-EM density map has secondary structural features at a medium resolution (4–6 Å), such as α helices or β sheets, one could build the protein backbone to obtain an overall shape of the molecule. Using cryo-EM density as a constraint, molecular dynamics (MD) simulations can be used to understand the movements of domains or residues, which are powerful approaches to studying the structural perturbation and energetics of biomolecules during their functional transitions [97]. Combined with 3D image classification, the MD trajectories derived from the cryo-EM densities could improve the understanding of the continuous motion of the target molecule.

### 7.1. Detecting Structural Variability Based on the Resolution Anisotropy

Because the conformational change of a biological molecule usually occurs regionally, the local resolution estimation could indicate the level of structural flexibility within the molecule [98,99]. If the atomic coordinates can be obtained from other experiments or the public depositories, such as Protein Data Bank (PDB), one can use the coordinates for an all-atom molecular simulation for training a model for dynamic information, which is implemented in the DEFMap program [100]. The structural variability of the target molecule can then be directly derived from the experimental cryo-EM image data. The advantage of this method is to reduce the computation cost dramatically compared to conventional all-atom molecular simulations because part of the dynamic information is obtained from the cryo-EM image data.

### 7.2. Molecular Dynamics Flexible Fitting (MDFF)

In most cases, single-particle reconstruction can only reach a medium resolution range (4–6 Å), which leaves spaces of uncertainty for modeling atomic coordinates. If a template of the atomic model exists, rigid-body fitting could be the first attempt to fit the model into the map, providing knowledge of domain organization in the target protein [101,102,103]. One can also use a cryo-EM density map as a geometric constraint and perform molecular dynamics flexible fitting (MDFF) to seek the best fit of the atomic model [104,105]. MDFF provides a theoretical framework for model building within a low-resolution cryo-EM map. The MDFF-fitted models built from cryo-EM maps of different states can be used to identify the domains or regions for protein conformational changes. Moreover, another molecular dynamics approach for cryo-EM model building is to optimize the real-space correlation between the model and experimental map based on the theoretical force fields, which results in a model close to the observation map without overfitting [106,107]. The approach is so-called correlation-driven molecular dynamics (CDMD), which has proven to be robust in modeling atomic coordinates in cryo-EM maps at a wide resolution range [106].

## 8. Time-Resolved Cryo-EM Studies

Because the protein particles are frozen simultaneously when prepared for cryo-EM imaging, the structures resolved in different states by single-particle cryo-EM do not contain any information in the time domain. Time-resolved cryo-EM aims to use cryo-EM to image or sample a reaction over a period of time until the reaction is in equilibrium. If the reaction is slow, the sampling time interval can be in minutes, allowing for the preparation of a frozen grid of a protein metastate [108,109]. However, if the reaction is fast, the frozen samples at different time points could be obtained using either microfluidic devices that allow the mixing of two reactants to occur or spraying/mixing methods in which a grid is covered with one reactant and the other is sprayed on [110]. The small volumes of samples at different reaction coordinates are ‘written’ or sprayed onto the grid for imaging, and the subsequent single-particle image classification and reconstruction will be utilized to analyze the molecular interactions or protein conformational changes. The recent development of time-resolved cryo-EM can be found in the following reviews and articles [110,111,112,113,114].

## 9. Interpretation of the Extracted Information for Biomolecular Perturbation

The molecular motions derived from cryo-EM data could be sampled in different 3D reconstructions from the above-mentioned image classification or data analysis. Because the molecules are all frozen simultaneously, the movements between distinct states may not have a causal connection. Structural variations between the reconstructions can be seen as conformational changes that occur with small energy transitions, and the proportions are weighted according to the Boltzmann distribution. During image processing, discrete motions could be observed to exhibit small subsets of dominant poses. However, continuous motions may arise when a molecule exhibits a high degree of flexibility and can adopt a large number of conformations with nearly equivalent weights in the Boltzmann distribution, which may not be trivial to analyze the data when the SNR is notoriously low.

The interpretation of protein motions learned from cryo-EM data will need to be validated using other biophysical or biochemical methods, such as cross-linking or molecular simulations. In particular, over-interpretation may be encountered when the motion does not relate to any function of the target protein.

## 10. Summary and Outlook

This review discusses different methods of analyzing the cryo-EM data heterogeneity and extracting structural variability information from the generated models. If a discrete distribution of the conformational states can be determined within the particle population, the validities of individual reconstructions could be verified using a standard approach, such as the gold standard FSC, with their data subsets. However, 3D reconstructions for continuous motion still require a robust validation method to be developed. Additionally, the conventional approach for particle curation may need to be revisited to prevent removing particle images that present the intermediate states. Cryo-EM opens a new route for analyzing structural variabilities of biomolecules and studying molecular dynamics, which provide insight into molecular mechanisms. The development of new data analysis methods will allow cryo-EM to facilitate the discovery of novel dynamic information about biomolecules.

## Data Availability

Not applicable.
